# Analysis of risk factors and a clinical prediction model for human cerebral echinococcosis in Ganzi region, China

**DOI:** 10.1093/braincomms/fcaf418

**Published:** 2025-10-28

**Authors:** Yong Zhong Chu, Wei Zhang, Yizhi Du, Zhuoma Danzhen, Liping Chen, Bingxi Lei

**Affiliations:** Department of Operations Management, People’s Hospital of Ganzi Tibetan Autonomous Prefecture, Kangding 626000, China; Department of Neurosurgery, People’s Hospital of Ganzi Tibetan Autonomous Prefecture, Kangding 626000, China; Department of Neurosurgery and Neuro–Oncology, State Key Laboratory of Oncology in South China, Collaborative Innovation Center for Cancer Medicine, Sun Yat-sen University Cancer Center, Guangzhou 510069, China; Department of Nursing, People’s Hospital of Ganzi Tibetan Autonomous Prefecture, Kangding 626000, China; Department of Biliary Duct, West China Hospital of Sichuan University, Chengdu 610041, China; Neurosurgery Department, Sun Yat-sen Memorial Hospital, Sun Yat-sen University, Guangzhou 510120, China

**Keywords:** human cerebral echinococcosis, encapsulated disease, Qinghai-Tibet Plateau, pathogenic factors, predictive modelling

## Abstract

The Qinghai-Tibet Plateau is one of the regions with the highest prevalence of cerebral echinococcosis worldwide. Investigating the risk factors for cerebral echinococcosis in Ganzi and developing an effective clinical risk prediction model is crucial for enhancing disease prevention and control in the region. Study participants were selected from the People’s Hospital of Ganzi Tibetan Autonomous Prefecture between January 2016 and December 2023. In this subject, 152 cases were diagnosed with cerebral echinococcosis (case group), while 580 cases were diagnosed with non-Echinococcus infection (control group). The chi-square test revealed significant differences in the component ratios for occupation, residential altitude, presence or absence of comorbidities with other sites of echinococcal infections, hypoproteinaemia, and tuberculosis (all *P* < 0.001). Logistic regression identified five variables—occupation, residential altitude, tuberculosis, hypovitaminosis, and combined infection in other sites—as risk factors for cerebral echinococcosis (*P* < 0.005). The predictive nomogram assigned the following scores: farmers (0), public officials or students (5.81), and herdsmen (60.624). The scores for the presence or absence of infections in other sites were 100 and 0, respectively. The scores for the presence or absence of hypoproteinaemia were 58.99 and 0, respectively. The scores for the presence or absence of tuberculosis were 54.65 and 0, respectively. The scores for altitudes ≤3000 m and >3000 m were 53.32 and 0, respectively. Internal validation of the nomogram's receiver operating characteristic curve using the Bootstrap method with 500 repeated samples showed the area under the curve was 0.920 (95% confidence intervals: 0.887–0.952). A confusion matrix was constructed using the true infection values, revealing a maximum Youden index of 0.76, sensitivity of 0.763, and specificity of 0.887. Internal validation using the Bootstrap method with 500 repeated samples showed that the calibration curve closely approximated the ideal curve, indicating that the model was well-calibrated. The Hosmer-Lemeshow goodness-of-fit test showed that *χ²* = 10.234, *P* > 0.05, further confirming that the model was well-calibrated. The decision curve analysis indicated that the model's best applicability for cerebral echinococcosis infection thresholds lies between 0.02 and 0.99. The nomogram model developed in this study for human brain echinococcosis infection demonstrated strong identification and predictive capabilities. The receiver operating characteristic curves and calibration plots confirmed the model’s high accuracy and consistency, further supporting its effectiveness. By identifying high-risk groups and protective factors for cerebral echinococcosis, the model offers a solid scientific foundation for the development of targeted prevention and control strategies.

## Teaser

Yongzhong et al. have studied the risk factors for cerebral echinococcosis in Ganzi and developed a predictive model to help identify high-risk individuals. They concluded that occupation, residential altitude, hypoproteinaemia, tuberculosis, and co-infections with other sites are key risk factors.

## Graphical Abstract

**Graphical Abstract:**
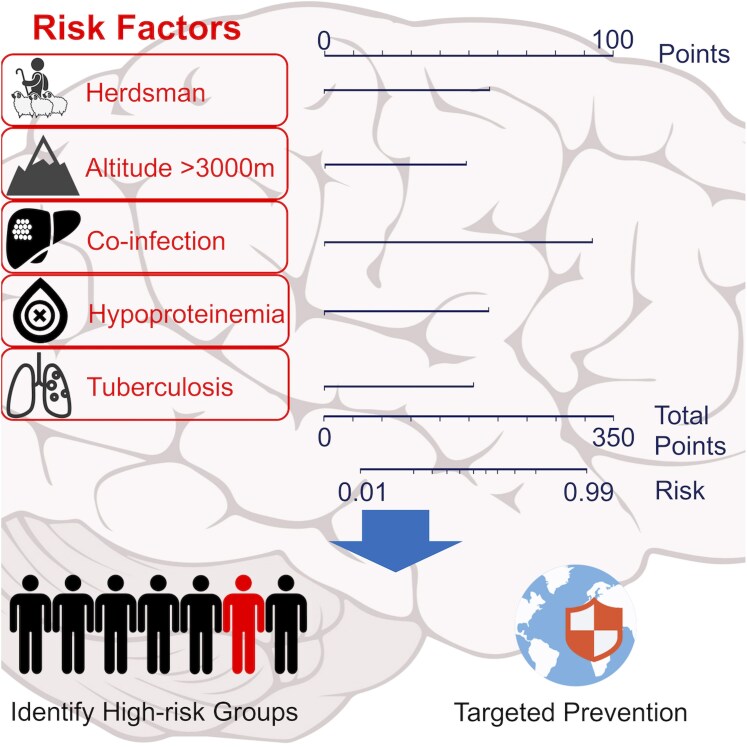

## Introduction

Echinococcosis (also known as bagworm disease) is a severe parasitic infection caused by the larvae of Echinococcus tapeworms, which is globally widespread and represents a significant threat to public health and livestock farming.^1,2^ This disease primarily consists of cysticercosis caused by fine-grained echinococcus tapeworms and vesicular echinococcosis caused by multihomed echinococcus tapeworms.^3,4^ It is highly endemic in the five northwestern provinces of China, particularly in the eastern and central regions of the Qinghai-Tibet Plateau. Despite a recent decline in the incidence of encapsular disease in China due to enhanced prevention and control measures, managing outbreaks and providing treatment in remote and underdeveloped regions continue to pose significant challenges.

The Qinghai-Tibet Plateau is one of the highest prevalence areas for echinococcosis worldwide, with cerebral echinococcosis often referred to as ‘parasitic cancer’ due to its mortality rate exceeding 90%.^5,6^ Despite the serious threat this disease poses to the health of local populations, research is lacking, particularly regarding risk factors for infection and the development of clinical prediction models.^7^ As a region with high echinococcosis prevalence, the incidence in Ganzi is influenced by various factors, including geographic environment, lifestyle habits, and economic conditions.^8^ Therefore, an in-depth analysis of the risk factors for cerebral echinococcosis infection in Ganzi, along with the development of an effective clinical risk prediction model, holds significant practical importance for enhancing disease prevention and control efforts and improving public health in the region.

## Materials and methods

### Eligibility criteria

This study is a cross-sectional study, which meets the ethical requirements of major prevention and control programmes such as the central financial subsidy for encapsular disease, and ethical approval was obtained from the unit. Informed consent was obtained from the patients themselves or their families for data collection.

The Inclusion criteria were: 1) permanent residency in Ganzi region of Sichuan, located on the Qinghai-Tibet Plateau; 2) clinical encounters at designated sentinel hospitals between January 2016 and December 2023; 3) confirmed diagnosis of cerebral echinococcosis supported by: epidemiological history (documented endemic-area exposure), characteristic clinical manifestations, typical CT/MRI findings, positive serological antibody test, and pathological verification; 4) age 8–75 years; 5) residence altitude ≥900 m; and 6) complete liver ultrasound, lung CT, serology, and biochemistry records.^9,10^ Patients were excluded for any of: 1) non-Ganzi residency or non-sentinel hospital records; 2) encounters outside January 2016–December 2023; 3) inconclusive cerebral echinococcosis diagnosis; 4) age <8 or >75 years; 5) long-term residence altitude <900 m; or 6) missing essential test results.^11,12^

### Study population and data collection

**Figure 1:**
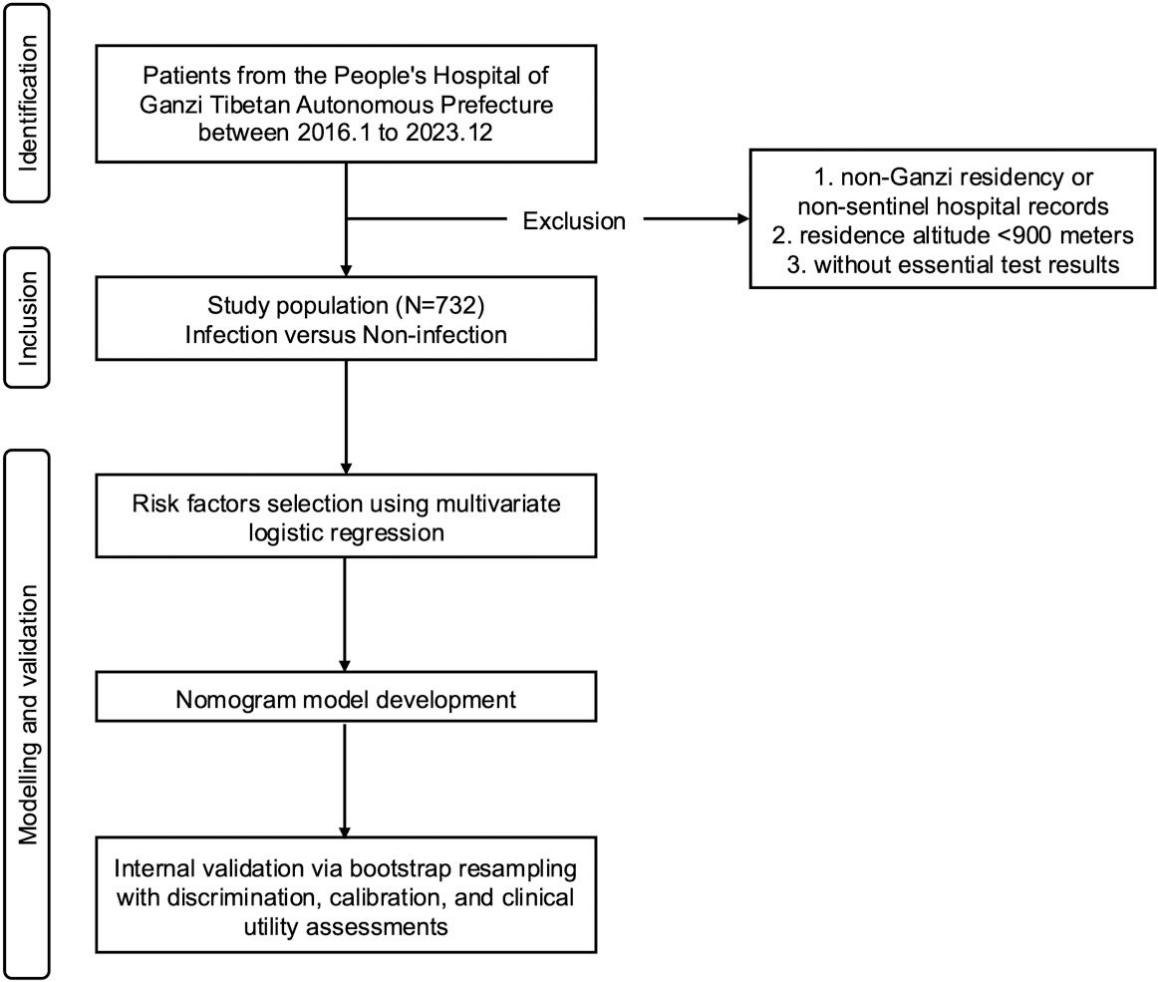
**Study design flowchart.** This study aimed to develop a model for predicting human cerebral echinococcosis risk by analysing patients from the People's Hospital of Ganzi Tibetan Autonomous Prefecture, China. The study cohort excluded individuals with non-Ganzi residency or incomplete clinical data.

Based on these predetermined criteria, we extracted records from the Hospital Information System (HIS), outpatient visit databases, and health check-up registries of sentinel hospitals in Ganzi. Finally, a total of 732 eligible subjects were enrolled and classified into: cerebral echinococcosis group (cases, *N* = 152) and non-Echinococcus infection group (controls, *N* = 580). For all subjects, we collected demographic variables including gender, age, occupation, residential altitude and comorbidity status including other-organ echinococcosis, hypoproteinaemia, tuberculosis. (Figure 1)

### Statistical analysis

The seven collected variables—gender, age, occupation, residential altitude, co-existing echinococcosis in other organs, hypoproteinaemia, and tuberculosis—were analysed using chi-square tests to compare differences between groups. Logistic regression analysis was then applied to identify risk and protective factors for cerebral echinococcosis infection.

The ‘rms’ package in R was employed to construct a predictive nomogram for human brain echinococcosis infection. Internal validation of the nomogram’s ROC curve was performed using the Bootstrap method with 500 repeated sampling iterations. A confusion matrix based on actual infection status was generated to determine the Youden index, sensitivity, and specificity. Calibration curves plotting predicted risk against actual frequency assessed model calibration, supplemented by internal validation via Bootstrap resampling (500 iterations). The Hosmer-Lemeshow goodness-of-fit test was conducted to further verify the model’s calibration. Finally, decision curves for the nomogram were constructed to analyse clinical applicability.

## Results

### Demographics

A total of 732 eligible patients were included in this study, of which 406 (55.46%) were male and 326 (44.54%) were female. The age distribution was as follows: 27 patients (3.69%) were in the 0–18 years age group, 445 patients (60.79%) were in the 19–49 years age group, and 260 patients (35.52%) were in the ≥50 years age group. In terms of occupation, 217 patients (29.64%) were herdsmen, 338 (46.17%) were farmers, and 177 (24.18%) were public officials or students. Regarding altitude, 255 patients (34.84%) lived at altitudes of 3000 m or below, while 477 patients (65.16%) resided at altitudes above 3000 m. Additionally, 129 patients (17.62%) were co-infected with echinococcosis in other parts of the body, 280 patients (38.25%) suffered from hypo-proteinaemia, and 102 patients (13.93%) had tuberculosis (Table 1).

**Table 1:** Clinical characteristics of individuals

Variables	Overall N *(%)*	Infection group N *(%)*	Non-infection group N *(%)*	*P value*
**Patients**	732	152 (20.77)	580 (79.23)	
**Sex**				0.001
Male	406 (55.46)	85 (55.92)	321 (55.34)	
Female	326 (44.54)	67 (44.08)	259 (44.66)	
**Age, year**				0.235
0–18	27 (3.69)	3 (1.97)	24 (4.14)	
19–49	445 (60.79)	100 (65.79)	345 (59.48)	
≥50	260 (35.52)	49 (32.24)	211 (36.38)	
**Occupation**				<0.001
Herdsman	217 (29.64)	120 (79.85)	97 (16.72)	
Farmers	338 (46.17)	20 (13.16)	318 (54.83)	
Public officials or students	177 (24.18)	12 (7.89)	165 (28.45)	
**Residential altitude, metre**				<0.001
≤3000	255 (34.84)	12 (7.89)	243 (41.9)	
>3000	477 (65.16)	140 (92.11)	337 (58.1)	
**Other sites of echinococcal infections**				<0.001
Yes	129 (17.62)	114 (75)	15 (2.59)	
No	603 (82.38)	38 (25)	565 (97.41)	
**Hypoproteinaemia**				<0.001
Yes	280 (38.25)	121 (79.61)	159 (27.41)	
No	452 (61.75)	31 (20.39)	421 (72.59)	
**Tuberculosis**				<0.001
Yes	102 (13.93)	77 (50.66)	25 (4.31)	
No	630 (86.07)	75 (49.34)	555 (95.69)	

### Imaging and pathology characteristics

**Figure 2:**
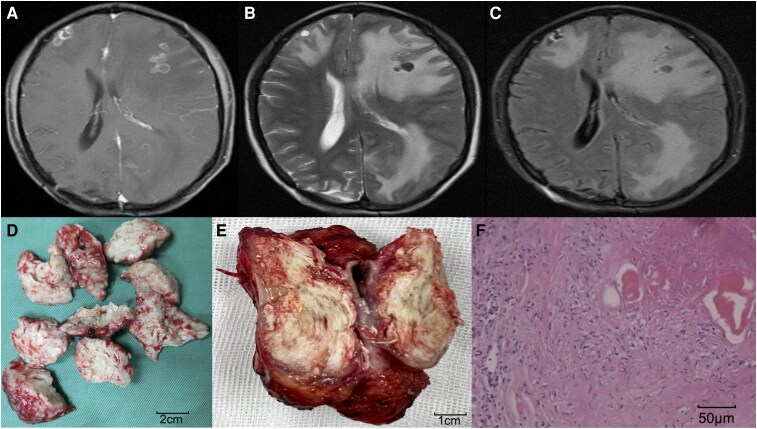
**Imaging and pathology images of cerebral echinococcosis. (A**) MRI T1-weighted contrast enhancement image shows multiple circular enhancement lesions located in the cerebral cortex, accompanied by extensive oedema around the lesions. (**B**) T2-weighted MRI and (**C**) T2 FLARE shows cerebral cystic echinococcosis is characterized by hypointensity signal with extensive oedema around the lesions. (**D**) Pathological specimen of cerebral cystic echinococcosis. (**E**) Pathological specimen of cerebral alveolar echinococcosis. (**F**) Pathological section manifestations of cerebral alveolar echinococcosis lesions (×200). FLAIR: Fluid Attenuated Inversion Recovery.

CT and MRI imaging of cerebral echinococcosis reveal characteristic findings. On non-contrast CT, the cystic lesions present as round or oval shapes with low-density content. Multiple adjacent cysts may indicate a potential rupture. The CT appearance of cystic echinococcosis resembles that of brain metastases, showing low-density lesions with marked surrounding oedema and often calcification in larger lesions. MRI clearly demonstrates cerebral cystic echinococcosis, displaying well-defined, round cystic lesions and allowing for the visualization of the number and distribution of daughter cysts within the mother cyst. MRI features of cerebral alveolar echinococcosis are characterized by multiple mixed-signal foci with significant perilesional oedema, and marked enhancement following contrast administration. Cerebral echinococcosis involves the formation of hydatid cysts with a two-layer membrane structure. The inner membrane, known as the hydatid cyst, is enclosed by an outer fibrous membrane derived from brain tissue. Between these two layers, blood vessels provide nutritional support. The inner membrane comprises a protective and elastic outer layer (the cuticular layer) and a germinal layer, which is the parasitic body itself. The germinal layer is responsible for producing brood capsules, daughter cysts, and scolices. (Figure 2).

### Cardinality test

The 732 patients were divided into infected (*N* = 152) and non-infected (*N* = 580) groups according to whether infection occurred or not, and the chi-square test was utilized to analyse the differences in demographics and the composition of comorbidities between the two groups. The results showed no significant differences in gender (*P* = 0.972) and age (*P* = 0.235) between the two groups. However, the differences in terms of occupation, altitude, co-infections at other sites, hypoproteinaemia, and tuberculosis were significant (all with *P* < 0.001). Specifically, the infected group had a much higher proportion of pastoralists (78.95%) compared to the non-infected group (16.72%), while the proportion of farmers (13.16% versus 54.83%) and public officials or students (7.89% versus 28.45%) was significantly lower in the infected group. The infected group also had a significantly higher proportion of individuals living at altitudes above 3000 m (92.11% versus 58.10%). Co-infections were much more common in the infected group, with 97.41% of patients reporting co-infections at other body sites compared to only 25.00% in the non-infected group. Furthermore, hypoproteinaemia was more prevalent in the infected group (72.59% versus 20.39%), as was tuberculosis (50.66% versus 4.31%) (Table 2).

**Table 2:** Univariate and multivariate analysis

Variables	Univariate analysis		Multivariate analysis	
	OR (95% CI)	P value	OR (95% CI)	P value
**Age, year**				
0–18	1.000 (Reference)		1.000 (Reference)	
19–49	2.319 (0.684–7.860)	0.177	1.082 (0.177–6.633)	0.932
≥50	1.858 (0.538–6.419)	0.327	1.485 (0.226–9.782)	0.681
**Sex**				
Male	1.000 (Reference)		1.000 (Reference)	
Female	1.024 (0.714–1.467)	0.899	1.337 (0.691–2.588)	0.388
**Occupation**				
Herdsman	1.000 (Reference)		1.000 (Reference)	
Farmers	0.051 (0.030–0.086)	<0.001	0.169 (0.081–0.351)	<0.001
Public officials or students	0.059 (0.031–0.112)	<0.001	0.230 (0.085–0.620)	0.004
**Residential altitude, metre**				
≤3000	1.000 (Reference)		1.000 (Reference)	
>3000	8.412 (4.561–15.516)	<0.001	4.761 (1.898–11.943)	0.001
**Other sites of echinococcal infections**				
Yes	1.000 (Reference)		1.000 (Reference)	
No	113.000 (60.145–212.302)	<0.001	18.572 (7.992–43.159)	<0.001
**Hypoproteinaemia**				
Yes	1.000 (Reference)		1.000 (Reference)	
No	10.335 (6.692–15.962)	<0.001	5.872 (2.683–12.851)	<0.001
**Tuberculosis**				
Yes	1.000 (Reference)		1.000 (Reference)	
No	22.792 (13.665–38.014)	<0.001	5.154 (1.991–13.342)	0.001

Abbreviations: OR: odds rate; 95%CI: 95% confidence intervals.

### Multivariate analysis

**Figure 3:**
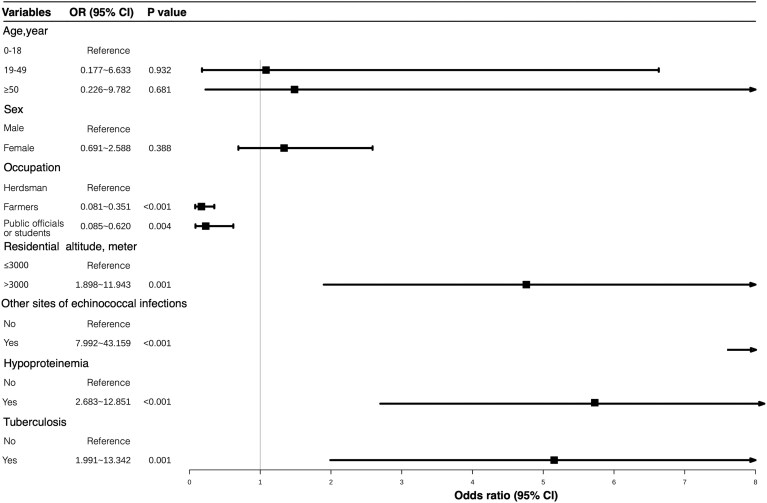
**Forest plot of univariate logistic regression analysis.** Odds ratios (OR) with 95% confidence intervals (95% CI) for demographic and clinical variables (*N* = 732). The OR and 95% CI were derived from univariate logistic regression models. The vertical line (OR = 1) indicates no significant association. Reference groups: occupation (herdsmen), altitude (≤3000 m), co-infection (absent), hypoproteinaemia (absent), tuberculosis (absent).

In this study, the following variables were included in multivariate logistic regression models: age (0–18 years = 1, 19–49 years = 2, ≥50 years = 3), sex (male = 1, female = 2), occupation (herdsmen = 1, farmers = 2, public officials or students = 3), altitude (≤3000 m = 1, >3000 m = 2), combined infections in other body sites (yes = 1, no = 2), hypo-proteinaemia (yes = 1, no = 2), tuberculosis (yes = 1, no = 2), and the presence of cerebral echinococcosis (yes = 1, no = 2) as the dependent variable. For categorical independent variables with ≥3 levels, corresponding dummy variables were created, using male as the reference category for gender, 0–18 years as the reference for age, herdsmen as the reference for occupation, and ≤3000 m as the reference for altitude (Table 2). The logistic regression results indicate that the *P*-values for occupation, residential altitude, tuberculosis, hypoproteinaemia, and co-infections in other body sites are all below 0.05, suggesting that these factors are significant predictors of cerebral echinococcosis. The specific effects were as follows: occupation—farmers and public officials or students were 0.169 times (95% CI: 0.081–0.351, *P* < 0.001) and 0.230 times (95% CI: 0.085–0.620, *P* = 0.004) more likely to develop cerebral echinococcosis compared to pastoralists, respectively; altitude—patients living at altitudes >3000 m were 4.761 times more likely to suffer from cerebral echinococcosis than those residing at or below 3000 m (95% CI: 1.898–11.943, *P* = 0.001); co-infections with other sites—patients with co-infections were 18.572 times more likely to develop cerebral echinococcosis compared to those without co-infections (95% CI: 7.992–43.159, *P* < 0.001); hypoproteinaemia—patients with hypoproteinaemia were 5.872 times more likely to develop cerebral echinococcosis than those without hypoproteinaemia (95% CI: 2.683–12.851, *P* < 0.001); tuberculosis—patients with tuberculosis were 5.154 times more likely to develop cerebral echinococcosis than those without tuberculosis (95% CI: 1.991–13.342, *P* = 0.001) (Fig. 3 and Table 2).

### Construction of a nomogram for the prediction of cerebral echinococcosis

**Figure 4:**
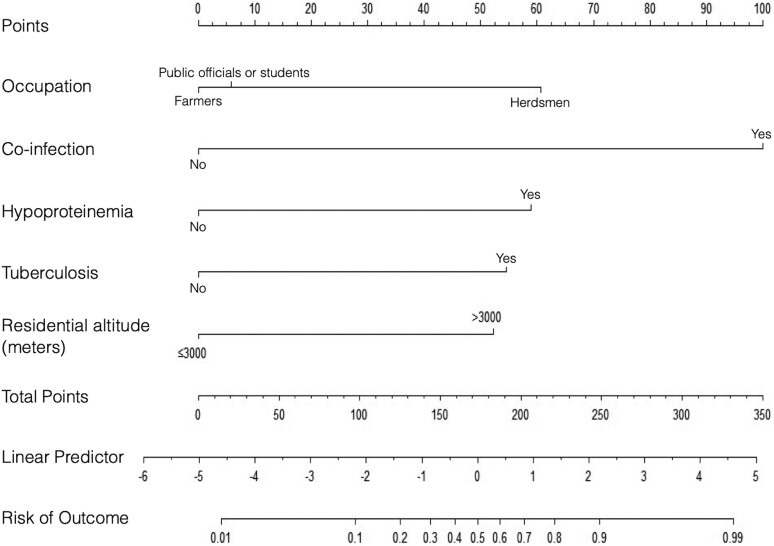
**Nomogram for predicting human cerebral echinococcosis infection.** The developed nomogram can predict the risk of cerebral echinococcosis infection. Individual scores are obtained by drawing a line from each variable value to the ‘Points’ line. The total risk score is then calculated by summing these individual risk scores. Finally, this cumulative score is used to estimate the probability or risk of cerebral echinococcosis infection.

The rms package in R was used to construct a predictive nomogram for cerebral echinococcosis, and multifactorial analysis identified occupation, co-infection with other body parts, hypoproteinaemia, tuberculosis, and altitude as significant factors influencing infection. Consequently, a nomogram for predicting cerebral echinococcosis was constructed with the dependent variable representing infection status, and the independent variables including occupation, co-infection with other body sites, hypoproteinaemia, tuberculosis, and altitude. A vertical line was drawn for each predictor variable, with the intersection of the vertical line and the corresponding score (Points) representing the value of that predictor. Total points are calculated by summing the individual scores, and the intersection of the total score with the risk axis represents the individual’s risk for echinococcus granulosus infection. The scores for occupation were as follows: farmers, public officials or students and herdsmen received scores of 0, 5.81, and 60.624, respectively. The scores for co-infection with other body sites were 100 and 0; for hypoproteinaemia, 58.99 and 0; for tuberculosis, 54.65 and 0; and for residential altitude (≤3000 m and >3000 m), 0 and 53.32, respectively (Fig. 4 and Table 3). The transformation from total points to infection risk follows the formula:


$$P=\frac{1}{1+e^{-x}}$$

where *P* represents the predicted probability of cerebral echinococcosis infection (range: 0–1), *e* is the base of the natural logarithm (≈2.718), and × represents the total points.

**Table 3:** Point for each variable of the nomogram

Variables	Option	Point
Occupation	Farmers	0
	Public officials or students	5.81
	Herdsman	60.624
Residential altitude, metre	≤ 3000	0
	> 3000	53.32
Other sites of echinococcal infections	Yes	100
	No	0
Hypoproteinaemia	Yes	58.99
	No	0
Tuberculosis	Yes	54.65
	No	0

### Validation of the nomogram for the prediction of cerebral echinococcosis

**Figure 5:**
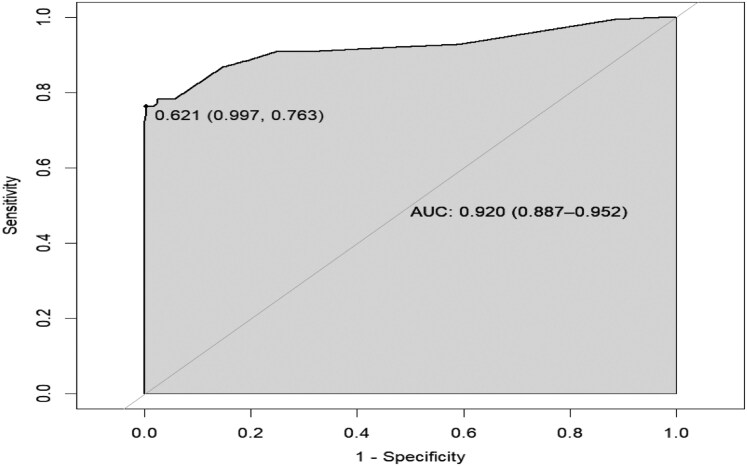
**ROC curve of the nomogram for predicting human cerebral echinococcosis infection.** Receiver Operating Characteristic (ROC) curve illustrating the specificity and sensitivity (1- specificity) of the nomogram. The area under the curve (AUC) was internally validated using the Bootstrap method with 500 repeated samples (*N* = 732). AUC = 0.920 (95% CI: 0.887–0.952).

**Figure 6:**
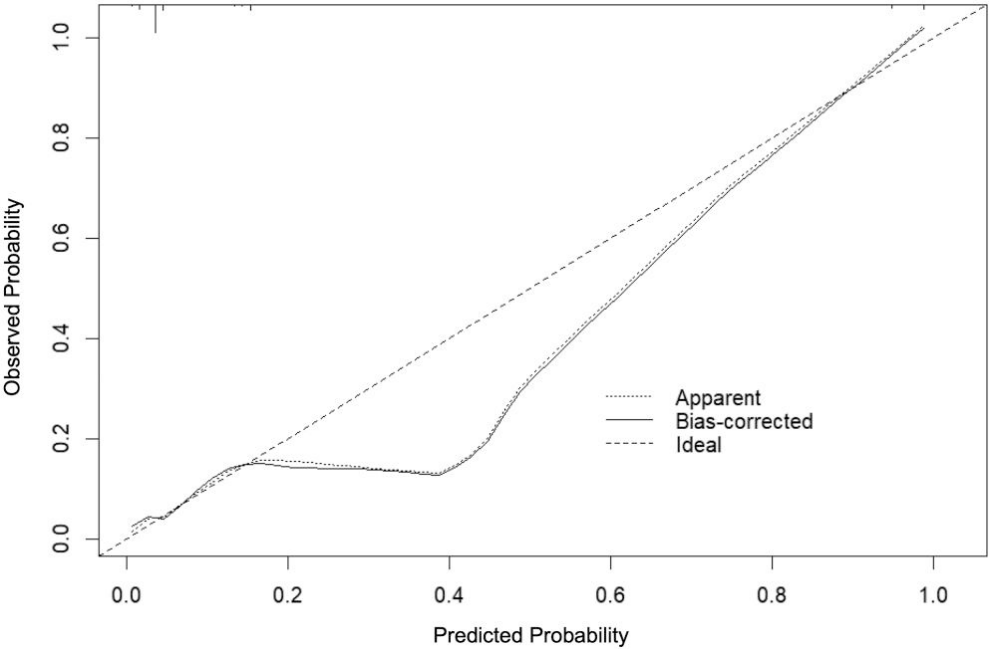
**Calibration curve of the nomogram for predicting human cerebral echinococcosis infection.** The calibration plot compares predicted probability (x-axis) against actual probability (y-axis). Internal validation was performed via Bootstrap resampling (500 repetitions; *N* = 732). The dotted line represents the ideal calibration, while the solid line reflects model performance. Hosmer-Lemeshow test: *χ²* = 10.234, *P* = 0.249.

Internal validation of the receiver operating characteristic (ROC) curve for the nomogram was performed by repeatedly sampling 500 times using the Bootstrap method. The results showed that the area under the curve (AUC) was 0.920, with a 95% confidence interval of 0.887–0.952. An AUC value greater than 0.9 indicated that the nomogram model had high accuracy and predictive value. A risk probability threshold of > 0.621 was used to predict cerebral echinococcosis, and a confusion matrix was constructed based on the true infection status. The Youden index reached a maximum value of 0.76. The sensitivity was 0.763, indicating that 76.3 out of 100 individuals who were truly infected with cerebral echinococcosis were correctly identified. The specificity was 0.887, meaning that 88.7% of individuals who were not infected were correctly identified. Calibration curves were plotted with predicted risk and actual frequency to assess the model's calibration. The ideal calibration curve was a 45° diagonal line. The findings showed that the calibration curve converged towards the ideal curve, indicating that the model is well-calibrated. Meanwhile, the Hosmer-Lemeshow goodness-of-fit test showed that *χ*^2^ = 10.234, *P* = 0.249 (> 0.05), further indicating that the model is well-calibrated. (Figure 5 and Fig. 6).

**Figure 7:**
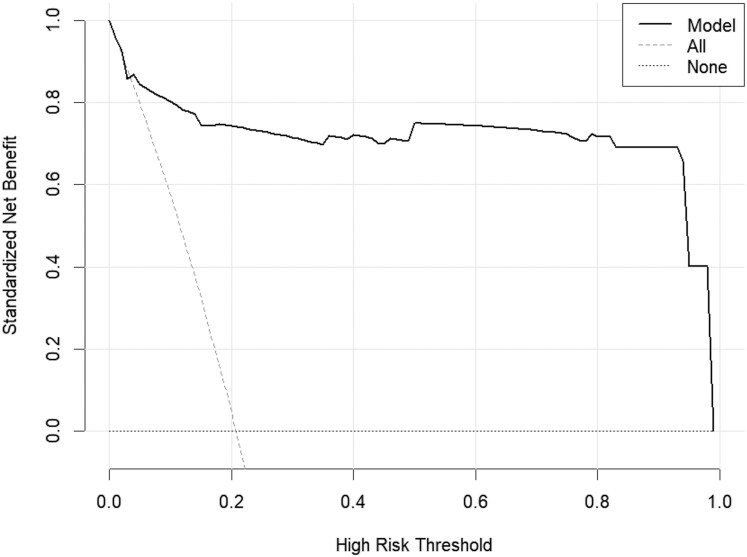
**Decision curve for the prediction of human cerebral echinococcosis infection using the nomogram.** Decision curve analysis (DCA) shows the net benefit of the nomogram across risk thresholds (x-axis) for the study cohort (*N* = 732). The dotted line assumes no infection in all patients; the dashed line assumes all patients are infected. The solid line represents the nomogram's clinical utility, with optimal applicability between thresholds of 0.02 and 0.99.

To compare the net benefits under different decision thresholds and select the optimal thresholds to maximize clinical benefits for patients, decision curves for the nomogram were constructed. The results showed that the nomogram model provided the best applicability for predicting cerebral echinococcosis infection at thresholds between 0.02 and 0.99 (Fig. 7).

## Discussion

### Occupation as a herdsman is significantly associated with the risk of cerebral echinococcosis

Compared to farmers, public officials or students, herdsmen are at a significantly higher risk of infection with cerebral echinococcosis, which is primarily attributed to frequent exposure to infectious agents, such as dogs and sheep, as well as living in pastoral environments with relatively inadequate sanitary conditions. Improving sanitary conditions and enhancing health education in pastoral areas is essential to reduce the risk of infection among herdsmen.^13^

### Environmental risk at high altitude

Individuals residing at altitudes exceeding 3000 m face a notably increased risk of contracting cerebral echinococcosis compared to those at lower altitudes. The combination of a cold climate, low oxygen levels, inadequate sanitation, and water sources susceptible to contamination by canine faeces at high altitudes contributes to an increased infection risk for residents.^14^ To mitigate this risk, it is recommended to enhance the development of sanitation infrastructure and implement rigorous water quality monitoring in high-altitude regions, while also promoting healthy lifestyle choices and water purification practices.^15,16^

### Effects of co-infection at other sites

Patients with co-infections at other sites of echinococcal infection were also significantly more likely to develop cerebral echinococcosis. This finding underscores the necessity of closely monitoring patients with multiple infection sites in clinical settings for early detection and intervention of potential cerebral infections.^17^ Furthermore, preventive strategies should focus on these high-risk groups, particularly patients with co-infections at other sites, with intensified surveillance and treatment.^18^

### Association with tuberculosis and hypoproteinaemia

Individuals with tuberculosis or hypoproteinaemia exhibit an increased susceptibility to cerebral echinococcosis. This may be attributed to the compromised immune system of these patients,^19,20^ which enhances their vulnerability to the parasite. Consequently, disease surveillance and nutritional support should be bolstered in these high-risk groups to mitigate infection rates.^9^

### Suggestions for prevention and control strategies

According to the WHO guidelines on echinococcosis prevention and control, the comprehensive promotion of echinococcosis control is crucial, requiring an integrated prevention strategy that predominantly targets infection sources while synergistically managing intermediate hosts and implementing standardized diagnosis and treatment of human cases.^21^

Praziquantel is currently the most effective anthelmintic available for controlling echinococcosis in carnivores. Administering praziquantel to dogs, which are the main infection sources of echinococcosis, are effectively manages their infections and significantly prevents the transmission of cystic echinococcosis to humans and livestock.^22,23^ Meanwhile, livestock immunization constitutes a core component of China's integrated control strategy for echinococcosis.^24,25^ A mandatory nationwide vaccination programme using the EG95 vaccine was initiated for sheep in echinococcosis-endemic regions under national policy directives and remains ongoing.^26,27^ Subsequently, pilot immunization trials extending EG95 vaccination to yaks were implemented in endemic areas.^28^

Beside domestic animals, wildlife also serves as a significant reservoir for echinococcosis transmission in Qinghai-Tibet Plateau. Wild canids, including foxes and stray dogs, are key definitive hosts. Molecular surveillance reports an overall prevalence of 5.5–5.6% in wild foxes and 15.2% in stray dogs.^29,30^ Small wild mammals usually act as essential intermediate hosts. Plateau pikas and voles are infected, while plateau zokors are confirmed as a novel intermediate host.^31,32^ Recently, Guo B *et al*. firstly reported echinococcosis in a wild boar in China, which means wild boars also serve as a transmitter for echinococcosis.^33^

By identifying high-risk groups and protective factors for cerebral echinococcosis, this study provides a robust scientific foundation for the development of targeted prevention and control strategies.^34,35^ Special attention and focused monitoring should be directed towards individuals who reside at high altitudes, work as herdsmen, have comorbidities involving other forms of organ echinococcosis, exhibit hypoproteinaemia, or have tuberculosis.^36^ Targeted preventive measures, including strengthening health education, improving living conditions and sanitation, enhancing the accessibility and quality of healthcare services, and conducting regular physical examinations and screenings, can facilitate early detection and treatment of potential infections, thereby reducing the risk of disease transmission.^37,38^

The findings and prediction model developed in this study are not only crucial for improving population health and preventing cerebral echinococcosis in the Ganzi region of the Qinghai-Tibet Plateau but also provide valuable insights for disease prevention and control in comparable regions.^39^ In regions with comparable geographic environments and lifestyles, these findings can assist local medical and health authorities in developing more scientifically grounded and effective prevention and control strategies to better safeguard public health.^17,40^

### Limitation

Firstly, this study is a retrospective case-control design, which may introduce recall bias and selection bias, potentially affecting the accuracy of the study results. Secondly, the study participants were sourced exclusively from sentinel hospitals in a specific region, which may not fully represent the broader regional population, leading to potential under-representation. Furthermore, the included risk factors may not be exhaustive, as potentially significant factors—such as genetic predispositions, detailed dietary habits, and personal hygiene practices—were not adequately considered.^2,4,6,37^

To address these limitations, future studies should be enhanced and refined. On one hand, prospective cohort studies could be conducted to more accurately elucidate the causal relationship between risk factors and diseases through long-term follow-up. Conversely, the scope of the study should be broadened to include a larger and more diverse sample from various medical institutions and communities, thereby enhancing the representativeness and generalizability of the findings. Additionally, further exploration of potential risk factors—such as genetic susceptibility and exposure to environmental pollutants—should be conducted to gain a comprehensive understanding of the disease's pathogenesis, thereby providing a more robust scientific foundation for the development of precise and effective prevention and control strategies.

In conclusion, this study has yielded valuable insights into the risk factors and the development of a predictive model for human cerebral echinococcosis infection in Ganzi, Qinghai-Tibet Plateau; however, there remains considerable potential for improvement and further exploration. Future research should build upon the foundation established by this study, aiming to further enhance and deepen the understanding of human cerebral echinococcosis, contribute more significantly to global disease prevention and control, and ultimately work towards reducing the disease burden and improving public health outcomes.^41^

## Supplementary Material

fcaf418_Supplementary_Data

## Data Availability

De-identified data can be provided upon a reasonable request to the corresponding author. All the codes generated or used in this work are supplied in the Supplementary material.

## References

[1] Liu L, Guo B, Li W, et al Geographic distribution of echinococcosis in Tibetan region of Sichuan province, China. Infect Dis Poverty. 2018;7(1):104. 30384860 10.1186/s40249-018-0486-4 PMC6214160

[2] Bai Y, Zhang Z, Jin L, et al Dynamic changes in the global transcriptome and MicroRNAome reveal Complex miRNA-mRNA regulation in early stages of the bi-directional development of *Echinococcus granulosus* protoscoleces. Front Microbiol. 2020;11:654. 32373094 10.3389/fmicb.2020.00654 PMC7188192

[3] Fu MH, Wang X, Han S, Guan YY, Bergquist R, Wu WP. Advances in research on echinococcoses epidemiology in China. Acta Trop. 2021;219:105921. 33878307 10.1016/j.actatropica.2021.105921

[4] Gottstein B, Wang J, Boubaker G, et al Susceptibility versus resistance in alveolar echinococcosis (larval infection with echinococcus multilocularis). Vet Parasitol. 2015;213(3–4):103–109. 26260407 10.1016/j.vetpar.2015.07.029

[5] Gong QL, Ge GY, Wang Q, et al Meta-analysis of the prevalence of echinococcus in dogs in China from 2010 to 2019. PLoS Negl Trop Dis. 2021;15(4):e0009268. 33798191 10.1371/journal.pntd.0009268 PMC8018629

[6] Li S, Chen J, He Y, et al Clinical features, radiological characteristics, and outcomes of patients with intracranial alveolar echinococcosis: A case series from Tibetan areas of sichuan province, China. Front Neurol. 2021;11:537565. 33519658 10.3389/fneur.2020.537565 PMC7843382

[7] Du G, Li Y, Wu P, et al Diagnosis, treatment, and misdiagnosis analysis of 28 cases of central nervous system echinococcosis. Chin Neurosurg J. 2021;7(1):30. 34020721 10.1186/s41016-021-00248-y PMC8139029

[8] He W, Wang LY, Yu WJ, et al Prevalence and spatial distribution patterns of human echinococcosis at the township level in Sichuan Province, China. Infect Dis Poverty. 2021;10(1):82. 34090538 10.1186/s40249-021-00862-z PMC8180058

[9] Yang G, Zhang Q, Tang G, et al Role of magnetic resonance spectroscopy and susceptibility weighted imaging in cerebral alveolar echinococcosis. Iran J Parasitol. 2015;10(1):122–127. 25904955 PMC4403531

[10] Yadav VK, Sudhakar SV, Panwar J. Pathognomonic MRI and MR spectroscopy findings in cerebral hydatid cyst. Acta Neurol Belg. 2016;116(3):353–355. 26525195 10.1007/s13760-015-0561-6

[11] Svrckova P, Nabarro L, Chiodini PL, Jäger HR. Disseminated cerebral hydatid disease (multiple intracranial echinococcosis). Pract Neurol. 2019;19(2):156–163. 30305379 10.1136/practneurol-2018-001954

[12] Li JM, Gan YJ, Niu XD, Wang TW, Yang Y, Mao Q. Atypical glioblastoma misdiagnosed as echinococcosis (a report of 1 case and review of literature). Journal of Clinical Neurosurgery. 2018;05:384–386.

[13] Chen S, Li N, Yang F, et al Medical treatment of an unusual cerebral hydatid disease. BMC Infect Dis. 2018;18(1):12. 29304756 10.1186/s12879-017-2935-2 PMC5756415

[14] Qucuo N, Wu G, He R, et al Knowledge, attitudes and practices regarding echinococcosis in Xizang Autonomous Region, China. BMC Public Health. 2020;20(1):483. 32293375 10.1186/s12889-020-8314-8 PMC7158018

[15] Ya-ming Y, Li-bo W, Fang-wei W, Xin-liu Y, Xiao-kun MA, Zun-wei DU. Survey of echinococcosis prevalence in area neigbouring Tibet in Yunnan. Dis Surv. 2014;29(1):52–55.

[16] Wang LY, Qin M, Liu ZH, et al Prevalence and spatial distribution characteristics of human echinococcosis in China. PLoS Negl Trop Dis. 2021;15(12):e0009996. 34962928 10.1371/journal.pntd.0009996 PMC8789093

[17] Wang Q, Yang L, Wang Y, et al Disease burden of echinococcosis in Tibetan communities-A significant public health issue in an underdeveloped region of western China. Acta Trop. 2020;203:105283. 31811863 10.1016/j.actatropica.2019.105283

[18] Bouomrani S . Cerebral hydatidosis: Exceptional and challenging form of neurohydatidosis. Microb Infect Dis. 2022;3:224–229.

[19] Blanc L, Gilleron M, Prandi J, et al *Mycobacterium tuberculosis* inhibits human innate immune responses via the production of TLR2 antagonist glycolipids. Proc Natl Acad Sci U S A. 2017;114(42):11205–11210. 28973928 10.1073/pnas.1707840114 PMC5651758

[20] Soeters PB, Wolfe RR, Shenkin A. Hypoalbuminemia: Pathogenesis and clinical significance. JPEN J Parenter Enteral Nutr. 2019;43(2):181–193. 30288759 10.1002/jpen.1451 PMC7379941

[21] WHO/OIE Manual on Echinococcosis in Humans and Animals: a Public Health Problem of Global Concern . Accessed 14 July 2025. https://iris.who.int/bitstream/handle/10665/42427/929044522X.pdf?sequence=1.

[22] Wei J, Cheng F, Qun Q, et al Epidemiological evaluations of the efficacy of slow-released praziquantel-medicated bars for dogs in the prevention and control of cystic echinococcosis in man and animals. Parasitol Int. 2005;54(4):231–236. 16231860 10.1016/j.parint.2005.06.002

[23] Andersen FL, Tolley HD, Schantz PM, Chi P, Liu F, Ding Z. Cystic echinococcosis in the Xinjiang/uygur autonomous region, people's republic of China. II. Comparison of three levels of a local preventive and control program. Trop Med Parasitol. 1991;42(1):1–10. 2052848

[24] Hua RQ, Du XD, He X, et al Genetic diversity of Echinococcus granulosus sensu lato in China: Epidemiological studies and systematic review. Transbound Emerg Dis. 2022;69(5):e1382–e1392. 35139582 10.1111/tbed.14469

[25] Yang YR, McManus DP, Huang Y, Heath DD. Echinococcus granulosus infection and options for control of cystic echinococcosis in Tibetan communities of Western Sichuan Province, China. PLoS Negl Trop Dis. 2009;3(4):e426. 19399162 10.1371/journal.pntd.0000426 PMC2668793

[26] Ministry of Agriculture and Rural Affairs, P.R. China . 2017. The national animal disease compulsory immunization program in 2017. Accessed 14 July 2025. https://www.moa.gov.cn/gk/ghjh_1/201703/t20170320_5530358.htm.

[27] Qian MB, Zhou XN. Walk together to combat echinococcosis. Lancet Infect Dis. 2018;18(9):946. 30152358 10.1016/S1473-3099(18)30466-3

[28] Ministry of Agriculture and Rural Affairs, P.R. China . (2021). The national animal disease compulsory immunization program in 2021. Accessed 14 July 2025. https://www.moa.gov.cn/govpublic/xmsyj/202101/t20210111_6359742.htm.

[29] Cai H, Zhang J, Zhang X, et al Prevalence of echinococcus Species in wild foxes and stray dogs in Qinghai Province, China. Am J Trop Med Hyg. 2021;106(2):718–723. 34781254 10.4269/ajtmh.21-0622 PMC8832913

[30] Zhang X, Fu Y, Ma Y, et al Brief report prevalence of Echinococcus species in wild foxes in parts of Qinghai Province, China. Vet Res Commun. 2023;47(2):947–952. 36333528 10.1007/s11259-022-10012-x

[31] Wang Z, Wang X, Liu X. Echinococcosis in China, a review of the epidemiology of echinococcus spp. Ecohealth. 2008;5(2):115–126. 18787915 10.1007/s10393-008-0174-0

[32] Ma J, Wang H, Lin G, et al Surveillance of Echinococcus isolates from Qinghai, China. Vet Parasitol. 2015;207(1–2):44–48. 25480467 10.1016/j.vetpar.2014.11.012

[33] Guo B, Cairen, Zhao L, et al First report of echinococcus granulosus genotype 1 in a wild boar (*Sus scrofa*) from China. Parasitol Res. 2024;123(6):236. 38856927 10.1007/s00436-024-08249-3

[34] Yu Q, Xiao N, Han S, Tian T, Zhou XN. Progress on the national echinococcosis control programme in China: Analysis of humans and dogs population intervention during 2004–2014. Infect Dis Poverty. 2020;9(1):137. 33008476 10.1186/s40249-020-00747-7 PMC7532088

[35] Senapati SB, Parida DK, Pattajoshi AS, Gouda AK, Patnaik A. Primary hydatid cyst of brain: Two cases report. Asian J Neurosurg. 2015;10(2):175–176. 25972961 10.4103/1793-5482.152109 PMC4421967

[36] Schmid M, Pendl G, Samonigg H, Ranner G, Eustacchio S, Reisinger EC. Gamma knife radiosurgery and albendazole for cerebral alveolar hydatid disease. Clin Infect Dis. 1998;26(6):1379–1382. 9636867 10.1086/516351

[37] Wen H, Vuitton L, Tuxun T, et al Echinococcosis: Advances in the 21st century. Clin Microbiol Rev. 2019;32(2):e00075-18. 30760475 10.1128/CMR.00075-18 PMC6431127

[38] Qian MB, Abela-Ridder B, Wu WP, Zhou XN. Combating echinococcosis in China: Strengthening the research and development. Infect Dis Poverty. 2017;6(1):161. 29157312 10.1186/s40249-017-0374-3 PMC5697071

[39] Budke CM, Casulli A, Kern P, Vuitton DA. Cystic and alveolar echinococcosis: Successes and continuing challenges. PLoS Negl Trop Dis. 2017;11(4):e0005477. 28426657 10.1371/journal.pntd.0005477 PMC5398475

[40] Yin J, Gongsang Q, Wang L, Li C, Wu X. Identification of vulnerable populations and knowledge, attitude, and practice analysis of echinococcosis in Tibet Autonomous Region of China. Environ Res. 2020;190:110061. 32810501 10.1016/j.envres.2020.110061

[41] Centers for Disease Control and Prevention . Accessed 14 July 2025. https://www.cdc.gov/

