# Supplementary material for: Analysis of risk factors and a clinical prediction model for human cerebral echinococcosis in Ganzi region, China
Source: Brain Commun. 2025 Oct 28;7(6):fcaf418. doi: 10.1093/braincomms/fcaf418 (PMC12669568; doi:10.1093/braincomms/fcaf418)
Supplement: fcaf418_Supplementary_Data [file fcaf418_supplementary_data.pdf]

Codes

R packages and data import

```
library(openxlsx)
library(rms)
library(CBCgrps)
library(pROC)
library(ResourceSelection)
library(rmda)
library(ggscidca)
library(autoReg)
data=read.xlsx("your_data_file.xlsx")
data$Sex=factor(data$Sex,labels=c("Male","Female"))
data$Age=factor(data$Age,labels=c("0-18","19-49","≥50"))
data$Occupation=factor(data$Occupation,labels=c("Herdsman","Farmers","Public
officials or students"))
data$Other_sites_of_echinococcal_infections=factor(data$Other_sites_of_echinococc
al_infections,labels=c("Yes","No"))
data$ Other_sites_of_echinococcal_infections=factor(data$ Other_sites_of_echinococ
cal_infections,levels=c("No","Yes"))
data$Hypoproteinemia=factor(data$Hypoproteinemia,labels=c("Yes","No"))
data$Hypoproteinemia=factor(data$Hypoproteinemia,levels=c("No","Yes"))
data$Tuberculosis=factor(data$Tuberculosis,labels=c("Yes","No"))
data$Tuberculosis=factor(data$Tuberculosis,levels=c("No","Yes"))
data$Residential_altitude=factor(data$Residential_altitude,labels=c("≤3000",">3000"
))
```

Clinical characteristics

```
Clinical characteristics=twogrps(data,"Diagnosis",cat.rd = 2,ShowStatistic = T)
write.csv(Clinical_characteristics $Table,"clinical_characteristics.csv")
```

Logistic regression analysis

```
fit_dan=glm(Diagnosis~Occupation+Other_sites_of_echinococcal_infections+Hypop
roteinemia+Tuberculosis+Residential_altitude,data=data,family=binomial())
autoReg::autoReg(fit_dan,uni=T,final=F)
```

Nomogram

```
dd=datadist(data)
options(datadist="dd")
fit1=lrm(Diagnosis~Occupation+Other_sites_of_echinococcal_infections+Hypoprotei
nemia+Tuberculosis+Residential_altitude,data=data,x=TRUE,y=TRUE)
nomogram=nomogram(fit1,fun=function(x){1/(1+exp(-x))},
                  fun.at = c(0.01,0.1,0.2,0.3,0.4,0.5,0.6,0.7,0.8,0.9,0.99),
```

```
                    funlabel = "Risks of Outcome",
                    lp=T,
                    conf.int = F
)
plot(nomogram)
```

ROC curve

```
pre_train=predict(fit1,data)
pre_train=1/(1+exp(-pre_train))
roc1=roc(data$Diagnosis,pre_train, ci = TRUE, boot.n=500)
plot(roc1,print.auc=TRUE,legacy.axes=T,print.thres=TRUE,auc.polygon=TRUE)
```

Calibration curve

```
call1=calibrate(fit1,method = "boot",B=500,estimates=TRUE)
plot(call1)
plot(call1,
     xlim = c(0,1),
     xlab = "Predicted Probability",
     ylab = "Observed Probability",
     legend = FALSE,
     subtitles = FALSE)
abline(0,1,col = "black",lty = 2,lwd = 2)
lines(call1[,c("predy","calibrated.orig")], type = "l",lwd = 2,col="black",lty = 3,pch
=16)
lines(call1[,c("predy","calibrated.corrected")], type = "l",lwd = 2,col="black",lty =
1,pch =16)
legend(0.68,0.5,
       c("Apparent","Ideal","Bias-corrected"),
       lty = c(3,2,1),
       lwd = c(2,2,2),
       col = c("black","black","black"),
       bty = "n",cex=1.2
)
```

Hosmer-Lemeshow goodness-of-fit test

```
hoslem.test(data$Diagnosis,pre_train,g=10)
```

Decision curve

```
baseline.model=decision_curve(Diagnosis~Occupation+Other_sites_of_echinococcal
_infections+Hypoproteinemia+Tuberculosis+Residential_altitude,family =
binomial('logit'),
                              data = data,confidence.intervals=F,
                              thresholds = seq(0,1,by=0.01))
plot_decision_curve(baseline.model,lty=c(1,2,3),curve.names =
```

```
"Model",cost.benefit.axis = F,col="black")
```

Forest plot

```
library(openxlsx)
library(forestplot)
library(forestploter)
options(scipen = 1)
dat=read.xlsx("your_data_file.xlsx")
dat=rbind(c("Variable", NA,NA,NA,"OR (95% CI)", "P value"),dat)
forestplot(dat[,c(1,5,6)],
           mean=dat[,2],
           lower=dat[,3],
           upper=dat[,4],
           graph.pos=4,
           xticks=c(0,1,2,3,4,5,6,7,8),
           clip=c(0,7),  is.summary=c(T,T,F,F,F,T,F,F,T,F,F,F,T,F,F,T,F,F,T,F,F,T,F,F),
           zero=1,xlim =c(0, 2),
           txt_gp=fpTxtGp (
             label=gpar(cex=0.8) ,ticks=gpar(cex=0.6)
           ),
           hrzl_lines=list("1" = gpar(lty=1,lwd=1.5),
                           "2" = gpar(lty=1,lwd=1.5)),
           col=fpColors ( box = 'black',
                          lines = 'black ' ,
                          zero = 'grey ),
           lwd.zero=1,
           boxsize = 0.35,
           lwd.ci=3,
           lty.ci=7 ,
           ci.vertices.height=0.09
)
```
